# Adherence to antiretroviral therapy among people living with HIV attending medication-assisted treatment clinics in Dar es Salaam, Tanzania: a cross-sectional study

**DOI:** 10.11604/pamj.2024.47.186.40498

**Published:** 2024-04-16

**Authors:** Johnson Dominic Mshangila, Hussein Hassan Mwanga, Magdalena Edes Shao, Daniel Joshua Msesa, Christopher Nyamtuma Mankaba

**Affiliations:** 1 Department of Official Development Assistance, Korea Foundation for International Healthcare, Dar es Salaam, Tanzania,; 2 Department of Environmental and Occupational Health, Muhimbili University of Health and Allied Sciences, Dar es Salaam, Tanzania,; 3 Department of Health Research, Zanzibar Health Research Institute, Zanzibar, Tanzania,; 4 Division of Health, Social Welfare, and Nutrition Services, Tarime Town Council, Mara, Tanzania

**Keywords:** People who inject drugs, adherence, antiretroviral therapy, human immunodeficiency virus, medication-assisted treatment

## Abstract

**Introduction:**

the prevalence of human immunodeficiency virus (HIV) among people who inject drugs (PWIDs) in Tanzania is estimated at 35%, significantly surpassing the 4.6% HIV prevalence of the general population. People who inject drugs living with HIV have been reported to exhibit lower adherence to antiretroviral therapy (ART), leading to increased rates of mortality, morbidity, and HIV transmission. This study assessed adherence to ART and associated factors among PWIDs in Dar es Salaam.

**Methods:**

this cross-sectional study involved 277 PWIDs living with HIV who attended MAT clinics in Dar es Salaam from May to July 2022. Antiretroviral therapy adherence was assessed using a validated one-month self-recall medication adherence scale, and associated factors were obtained through a structured questionnaire. Statistical analyses included chi-square tests, Fisher exact tests, and log-binomial regression. Data were analyzed using STATA version 15, with a p-value of <0.05 considered statistically significant.

**Results:**

this study found that 83% of the study participants had a high level of adherence to ART. Additionally, the results revealed that PWIDS who consume alcohol were less likely to have high adherence to ART (aPR 0.820). On the other hand, higher odds of ART adherence were observed among participants who had family support (aPR 1.028) and those who had adequate knowledge of ART benefits (aPR 1.285).

**Conclusion:**

the government and development partners should implement novel interventions such as alcohol reduction programs, ART education, and expanded HIV community outreach services. These interventions have the potential to improve ART adherence and reduce HIV transmission among PWIDs.

## Introduction

People who inject drugs (PWIDs) are among the most vulnerable populations at risk of acquiring HIV [1]. Worldwide, there are approximately 12 million PWIDs, of whom 1.6 million are living with HIV [1,2]. In Africa, there are around 630,000 PWIDs, with a 13.6% prevalence of HIV among them. In Tanzania, the prevalence of HIV among PWIDs is 35%, and it is estimated that PWIDs are 29 times more likely to contract HIV than the general population, which has a 4.6% prevalence of HIV [1,3,4].

The majority of studies on adherence to antiretroviral therapy (ART) have focused on vulnerable groups such as sex workers, men having sex with men (MSM), and adolescents while leaving behind PWIDs, who face a higher risk of contracting HIV compared to other populations. In Tanzania, there is limited information on factors affecting ART adherence among PWIDs living with HIV who are attending Medication-assisted Treatment clinics (MAT). This highlights a significant knowledge gap that needs to be addressed and assessed. Like other ART users, PWIDs must achieve at least 95% adherence to ART to reduce HIV-related mortality and morbidity, as well as prevent the transmission of HIV to other individuals in the community [2,3,5]. Understanding this level of ART adherence and factors associated with adherence to ART among people who inject drugs in our context will help to increase adherence to ART and reduce treatment failure as well as antiretroviral drug resistance among PWIDS on ART at MAT clinics across the country [6-8].

We conducted a cross-sectional clinical-based study among PWIDs living with HIV in Dar es Salaam, Tanzania, to examine adherence levels to ART and identify the factors associated with ART adherence. Our primary hypothesis was that various socio-demographic, socio-behavioral, and patient-related factors would significantly influence adherence to ART. By investigating these factors, we sought to provide valuable insights for designing targeted interventions and identifying populations that may benefit from additional support in improving ART adherence.

## Methods

**Study design:** a cross-sectional analytical study design was conducted to determine the level of adherence and factors associated with adherence to ART among PWIDs living with HIV attending MAT clinics.

**Setting:** this study was conducted at Muhimbili National Hospital, Temeke Regional Referral Hospital, and Mwananyamala Regional Referral Hospital in the Dar es Salaam region of Tanzania from May 2022 up to July 2022. Participants met in a private room with an interviewer who was then asked for consent and then administered research questions in an interview schedule form orally in Swahili language using Google Forms as a means of data collection. In addition, all questions in the interview tool were made mandatory to avoid missing any information from participants. A pretest was conducted with ten PWIDs living with HIV from the Mwananyamala Regional Referral Hospital MAT clinic. The questionnaire was administered by the research team to assess its accuracy and ability to address the study's research questions. The pretest participants were not included in the main study.

**Participants:** the study participants were: (i) people who inject drugs, (ii) those aged 18 years old and above, (iii) those living with HIV, (iv) those who were attending MAT clinics, and (v) received ART for six months and above at Muhimbili National Hospital, Temeke Regional Referral Hospital, and Mwananyamala Regional Referral Hospital. Due to the small size of the study population, no sampling technique was used, but instead, all PWIDS living with HIV who met the inclusion criteria were recruited.

**Variables:** the dependent variable in this study was adherence to antiretroviral therapy among PWIDs living with HIV. The study also examined several exposure variables, including socio-behavioral factors such as alcohol use, having a supporter, and disclosure of information. Furthermore, patient-related factors, such as knowledge of the benefits of ART and compliance with ART instructions, were taken into account. In addition to these variables, age and monthly income were identified as potential confounders, recognized for their potential influence on adherence to ART among people who inject drugs living with HIV.

**Data source/measurement:** data on adherence to ART was collected from PWIDs living with HIV through the use of a validated one-month self-recall medication adherence scale measurement questionnaire while data on demographic characteristics, socio-behavioral factors, and patient-related factors were measured using a structured questionnaire. The study measurement tool had a Cronbach Alpha coefficient of 0.8018, an item-rest correlation ranging from 0.5383 to 0.698, and an average variance of 0.1619. These robust statistical measures validate the instrument's reliability and effectiveness in assessing the intended measurement of interest.

**Bias:** the study's susceptibility to recall bias was effectively addressed through the utilization of standardized data collection tools. Validated questionnaires and structured questions were employed to minimize variability and enhance the accuracy of participant responses.

**Study size:** the study enrolled a total sample size of 277 PWIDs living with HIV. The determination of the sample size took into account various factors, including the estimated prevalence of adherence to antiretroviral therapy, which was found to be 63% based on findings from a previous study [9]. Additionally, a 95% confidence interval, a 6% margin of error, and a non-response rate of 10% were considered during the determination process.

**Quantitative variables:** the primary outcome of this study was adherence to ART among PWIDs living with HIV. Adherence was assessed using a validated one-month self-recall medication adherence scale, which comprised eight questions. Each question had a dichotomous response of either “YES” or “NO.” The first seven questions were answered with “YES” or “NO,” while the eighth question utilized a five-point Likert scale response. To avoid potential bias towards saying “YES,” the tool was designed in a way that questions 1 to 4 and 6 to 8 were reverse coded, while question 5 was coded normally (with “Yes” assigned a value of 1 and “No” assigned a value of 0). The obtained scores were recorded for questions 1 to 4 and 6 to 8, while question 5 remained in its initial coded form. Subsequently, the scores were summed to obtain a total score. Scores below 6 were classified as indicating low adherence to ART, while scores of 6 or higher were classified as indicating high adherence to ART. This adherence level was then expressed in terms of proportion. Exposure variables, such as demographic factors, were included in this study. These variables were categorized as follows: 1) age group: participants' age was categorized into five groups (20-29 years, 30-39 years, 40-49 years, 50-59 years, and 60-69 years). This categorization aimed to capture different age stages and potential variations in adherence to ART across various age ranges; 2) sex: participants' sex was categorized as male or female. This categorization was done to account for potential gender-based differences in ART adherence; 3) education: education levels were classified into four groups (primary school, secondary school, university, and none attended school). This categorization was intended to assess the influence of educational attainment on ART adherence since different educational levels may influence knowledge and decision-making processes; 4) occupation status: participants' occupation status was categorized as employed, self-employed, or jobless. This categorization was based on the understanding that occupation status may reflect factors such as income stability, and time availability for healthcare-related activities; 5) income status: income status was categorized as follows: less than 200,000 Tsh, (200,000-500,000) Tsh, and above 500,000 Tsh. This categorization aimed to explore the impact of income levels and how can influence ART adherence.

Socio-behavioral factors related to antiretroviral therapy were assessed through the following measures: firstly, the presence of a patient supporter was assessed by asking participants whether they received assistance in using ART, with the response options of “YES” or “NO.” Secondly, participants were asked about their alcohol consumption in the past weeks, and they could respond with a “YES” or “NO” regarding their alcohol use during that period. Lastly, participants were asked about the disclosure of information about ART, specifically who they shared this information with. They were given options such as spouses, close relatives, and friends, and they could select one or more of these options to indicate the individuals they shared ART-related information with. These measures aimed to capture important socio-behavioral aspects of ART utilization, the presence of patient supporters, alcohol consumption patterns, and the disclosure of ART information within the participants' social network. Patient-related factors, such as client ART knowledge and compliance with dispenser instructions on ART use, were assessed in this study. Antiretroviral therapy (ART) knowledge was categorized using a structured adopted tool with a Likert scale, with levels of “low,” “moderate,” and “adequate” representing different levels of ART knowledge. Compliance with dispenser instruction on ART use was categorized as either “yes” or “no,” indicating whether participants followed the ART dispenser instruction.

**Statistical methods:** the data collection process involved the utilization of Google Forms for secure storage and efficient management. Subsequently, the data was downloaded from Google Forms in the form of a Microsoft Excel file. The acquired data was then imported into Stata version 15 for comprehensive cleaning, consistency checking to address missing values, and evaluating the normal distribution of all variables before conducting the analysis. For the first objective, the descriptive analysis was done to analyze the general characteristics of the participants and the proportion of adherence to antiretroviral therapy among people who inject drugs attending MAT clinics.

For the second and third objectives, bivariate analysis was done using the Chi-squared test or Fisher´s exact test to evaluate the association between the independent variables and adherence to ART. In bivariate analyses, all independent variables associated with adherence to ART at p-value <0.20 were entered into multiple log-binomial regression model adjusted for age and monthly income. The results were reported as prevalence ratios (PR) and adjusted prevalence ratios (aPR) with 95% confidence intervals (the decision to use prevalence ratios was based on the fact that the prevalence of the outcome of interest was above 10%). All variables with small p-values (P <0.2) were added into a model one after another using forward stepwise selection until the final model was generated.

**Ethical approval:** the study requested ethical approval (Ref. No. Da.282/298/01.C) from the Institutional Review Board (IRB) of Muhimbili University of Health and Allied Sciences. The signed approval, introduction letters, fully researched proposal, and commitment letters were presented to the hospital management to request permission to access their facilities and information. The MAT numbers were used to protect the identities of participants and ensure their anonymity. Participant information was treated as strictly confidential and was not used for any unintended purposes.

## Results

The study included a total of 277 participants with a mean age of 40 years (SD ± 8.18378), majority of the participants were male (74%), cohabiting (39%), unemployed (61%), and primary school education levels (55%) and low income (77%). Of the 277 participants, 83% demonstrated high adherence to antiretroviral therapy. Table 1 provides an overview of the demographic characteristics and adherence level of participants. Association between socio-behavioral factors and adherence to antiretroviral therapy among people who inject drugs. A strong and statistically significant association was observed between alcohol consumption and adherence to ART (p-value < 0.001). Furthermore, a strong association was observed between family support and adherence to antiretroviral therapy (p-value < 0.001). Additionally, disclosing information about one's health condition (HIV) to others was found to be strongly associated with adherence to antiretroviral therapy (p-value 0.001). Table 2 presents a summary of socio-behavioral factors associated with adherence to antiretroviral therapy.

**Table 1:** socio-demographic characteristics of the study participants according to the level of adherence to antiretroviral therapy

Variables	Category	Overall n (%)	Low adherence n (%)	High adherence n (%)	P-value
**Adherence Level**			48 (17)	229 (83)	
**Sex**	Male	206 (74)	33 (16)	173 (84)	0.327*
	Female	71 (26)	15 (21)	56 (79)	
**Age group (years)**	20-29	28 (10)	4 (14)	24 (86)	0.210**
	30-39	84 (30)	16 (19)	68 (81)	
	40-49	130 (47)	18 (14)	112 (86)	
	50-59	32 (12)	10 (31)	22 (69)	
	60-69	3 (1)	0 (0.00)	3 (100)	
**Marital status**	Single	57 (21)	11 (19)	46 (81)	0.558**
	Married	54 (19)	13 (24)	41 (76)	
	Separated	48 (17)	8 (17)	40 (83)	
	Co-habit	108 (39)	15 (14)	93 (86)	
	Widow	10 (4)	1 (10)	9 (90)	
**Education level**	Primary school	152 (55)	26 (17)	126 (83)	0.964**
	Secondary school	73 (26)	14 (19)	59 (81)	
	University/college	8 (3)	1 (12)	7 (88)	
	None	44 (16)	7 (16)	37 (84)	
**Occupation status**	Employed	18 (7)	5 (28)	13 (72)	0.373**
	Self-employed	90 (32)	17 (19)	73 (81)	
	Not employed	169(61)	26 (15)	143 (85)	
**Monthly Income**	<200,000 Tsh	212(77)	32 (15)	180 (85)	0.173**
	200,000-500,000 Tsh	59 (21)	15 (25)	44 (75)	
	> 500,000 Tsh	6 (2)	1 (17)	5 (83)	
**Live on the street at night**	No	269 (97)	40 (15)	229 (85)	<0.001**
	Yes	8 (3)	8 (100)	0 (0.00)	

* Chi-squared test; **Fisher's exact test

**Table 2:** socio-behavioral factors associated with adherence to antiretroviral therapy

Variables		Low adherence n (%)	High adherence n (%)	P-value
		48 (17)	229 (83)	
**Alcohol use**	No	15 (9)	143 (91)	<0.001*
	Yes	33 (28)	86 (72)	
**Family support**	No	33 (33)	68 (67)	<0.001*
	Yes	15 (9)	161 (91)	
**Disclosure of information**	Spouse	2 (6)	33 (94)	<0.001**
	Close friend	0 (0)	14 (100)	
	Close relative	5 (3)	147 (97)	
	None	41 (54)	35 (46)	

* Chi-squared test; **Fisher's exact test

Association between patients-related factors and adherence to antiretroviral therapy among people who inject drugs. Among the participants in the study, no significant association was found between knowing the benefits of regular ART and adherence to ART (p-value 0.077). However, to further explore potential confounding factors that may have influenced this association, a multiple regression analysis was conducted. Compliance with the dispenser instructions on ART was found to be not statistically significance with (p-value 0.508). Table 3 presents a summary of patients' related factors associated with adherence to antiretroviral therapy.

**Table 3:** patient-related factors associated with adherence to antiretroviral therapy

Variables	Category	Low adherence n (%)	High adherence n (%)	P-value
**Compliance with dispenser instruction on ART**	No	4 (24)	13 (76)	0.508**
	Yes	44 (17)	216 (83)	
**Knowledge on ART**	Low	6 (29)	15(71)	0.077*
	Moderate	11 (19)	46 (81)	
	Adequate	23 (12)	164 (88)	

* Chi-squared test; **Fisher's exact test

The bivariate and multivariate analyses for factors associated with adherence to antiretroviral therapy are shown in Table 4. In bivariate analysis, participants who recently were taking alcohol had a lower prevalence of having high adherence to antiretroviral therapy compared to participants who were not taking alcohol (PR, 0.798 (95% CI, 0.707-0.902) and in multivariate analysis similarly found that alcohol intake was statistically significant (aPR 0.820, 95% CI 0.728-0.923).

**Table 4:** factor associated with adherence to antiretroviral therapy in bivariate and multivariate analysis

Variables		Crude prevalence ratios		Adjusted prevalence ratios	
		**PR**	**95% CI**	**aPR**	**95% CI**
**Alcohol use**	No	1		1	
	Yes	0.798	0.707-0.902**	0.820	0.728-0.923*
**Family support**	No	1		1	
	Yes	1.431	1.245-1.644**	1.028	0.906-1.165
**Disclosure of information**	Spouse	1		1	
	Close friend	1		1	
	Close relative	1.026	0.941-1.119	1.007	1.006-1.007 **
	None	0.488	0.378-0.631**	0.481	0.377-0.614**
**Knowledge on ART**	Low	1		1	
	Moderate	1.130	0.838-1.523	1.15	0.848-1.553
	Adequate	1.228	0.932-1.618	1.285	0.970-1.702

cPR- crude prevalence ratio, aPR - adjusted prevalence ratio, CI =confidence interval, Ref: reference; adjusted for age and monthly income; *p-value < 0.01; **p-value <0.001

Participants who were receiving support from family on the use of ART had a higher prevalence of having high adherence to antiretroviral therapy than those participants who declared not receiving family support on the use of antiretroviral therapy (PR =1.431; 95% CI: 1.245-1.644). However, after multivariate analysis, the family support was not significant (aPR =1.028; 95% CI: 0.906-1.165). Also, participants who declared they are not disclosure their health condition to anyone had a low prevalence of having high adherence to antiretroviral therapy compared to those who disclosure information to their close people (PR= 0.488; 95% CI: 0.378-0.631), in multivariate analysis similarly found that disclosure of health information was statistically significant (aPR 0.481, 95% CI 0.377-0.614). Furthermore, on bivariate analysis, participants who had adequate knowledge of ART had more prevalence of being adherent to antiretroviral therapy than those who participants with low knowledge and it was not statistically significant (PR = 1.228, 95% CI: 0.932-1.618) same as multivariate analysis (aPR = 1.285; 95% CI: 0.970-1.1702).

## Discussion

This study revealed that 83% of PWIDs living with HIV demonstrated high adherence to ART. While this adherence level is encouraging, it is important to acknowledge that it falls below the recommended threshold of 95% adherence to ART [1]. Compared with other relevant studies, the adherence level reported in this study was lower than the study conducted in Tanzania in 2018 on adherence to ART among people who inject drugs attending opioid treatment programs, which reported an adherence rate of 97.2% [10]. Moreover, studies conducted in Pakistan in 2021 and Indonesia in 2019 reported adherence rates of 55% and 74.5% respectively among people who use drugs, both of which are lower than the adherence level reported in our study [6,9].

The disparities in ART adherence across those different studies may be attributed to the variations in adherence assessment methods such as pill count, structured self-report, or electronic monitoring systems and the definition of adherence level. Nevertheless, this finding underscores the need for further interventions and strategies to enhance adherence among this key vulnerable population. Tailored approaches should be developed to address the unique challenges faced by PWIDs in maintaining optimal adherence to ART.

Socio-behavioral factors such as alcohol consumption, family support, and information disclosure have a significant impact on adherence to ART among PWIDs. Regarding alcohol consumption, participants who reported consuming alcohol during their treatment demonstrated lower adherence to ART compared to non-drinkers. This finding aligns with a similar study in Estonia, which found hazardous alcohol use to be strongly associated with decreased ART adherence. Individuals who consumed alcohol were 55% more likely to have poor adherence compared to non-drinkers [11]. The similarities are likely due to the nature of substance use, including alcohol consumption, which can negatively impact cognitive abilities, decision-making processes, and self-care practices, leading to decreased adherence to ART among PWIDs. Addressing alcohol consumption is crucial for improving ART adherence among PWIDs.

Family support also played a pivotal role in ART adherence among PWIDs. Participants who received reliable family support exhibited higher levels of adherence to ART. This finding is consistent with a study in Kenya that highlighted the positive association between family support and ART adherence [12]. Furthermore, a systematic review encompassing low- and middle-income countries underscored the importance of support services, including primary care and family support, in improving ART adherence among PWIDs [13]. The similarities observed between this study and other studies can be explained by the significant impact of supportive family members in providing practical assistance, emotional encouragement, and accountability, leading to improved ART adherence. Integrating family support interventions into ART treatment programs for PWIDs is crucial based on these findings and existing literature.

Disclosure of health information plays a role in fostering adherence to ART among PWIDs; participants who chose not to disclose their health condition to anyone showed lower ART adherence compared to those who disclosed their status to close contacts. A study conducted in Estonia supported this finding, indicating that health information disclosure was associated with improved adherence to ART [11]. This similarity can be explained as disclosing one's health status fosters a support network that provides practical assistance and emotional support, leading to better adherence. These results emphasize the importance of interventions aimed at creating a supportive environment within the community, as disclosure of HIV and ART status facilitates open communication regarding treatment adherence, medication reminders, and healthcare appointment assistance. Ultimately, such interventions can lead to improved ART adherence and better health outcomes for PWIDs.

Knowledge of the benefits of adhering to ART plays a part in improving ART adherence among PWIDs. Individuals with an adequate level of knowledge about the benefits of ART demonstrate higher adherence compared to those with limited knowledge. This finding aligns with systematic reviews conducted in low- and middle-income countries, consistently showing a positive correlation between knowledge about the benefits of ART and high adherence to antiretroviral therapy [14,15].

Understanding the benefits of ART empowers individuals, enabling them to make informed decisions about their treatment and take personal agency and responsibility for their health. Adequate knowledge about the benefits of ART enhances patients' comprehension of the importance of adherence and the impact it can have on their health outcomes. It also facilitates active engagement in discussions with healthcare providers, seeking support as needed, and addressing any concerns or misconceptions related to ART. In light of these findings, interventions aimed at promoting ART adherence should prioritize comprehensive education programs. These programs should focus on enhancing patients' knowledge about the benefits of ART, addressing misconceptions, and providing ongoing support to improve their adherence to ART.

This study acknowledges several limitations. Firstly, the inclusion criteria restricted the sample to PWIDs living with HIV who attended the MAT clinic on the day of the interview. As a result, individuals who had missed doses of methadone or buprenorphine, were ill, or were enrolled in the new take away dose (TAD) program were not included, potentially introducing bias and limiting the comprehensiveness of the findings. Furthermore, due to time constraints, follow-up with participants was not possible, which could have provided valuable longitudinal insights into their adherence behaviors. The absence of longitudinal data may limit the ability to capture the dynamic nature of adherence patterns and the influence of various factors over time.

The study's focus on PWIDs living with HIV recruited from MAT clinics, which have a high population in Tanzania, improves generalizability to similar low- and middle-income settings. However, due to potential differences in settings and population characteristics, caution is advised when applying the findings to PWIDs who do not attend MAT clinics, particularly those in drug rehab centers. Future replication studies should include diverse samples from different regions of the country to provide a broader understanding of the research's generalizability and applicability. This will strengthen the overall validity and robustness of the findings.

## Conclusion

Improving adherence to ART among PWIDs living with HIV is crucial as it currently falls short of the optimal adherence level of 95% recommended by WHO, UNAIDS (Joint United Nations Programme on HIV/AIDS), and the national targets set by the Tanzania Ministry of Health. To address this gap, a collaborative effort between the government and implementing partners is very essential. Key interventions should prioritize addressing alcohol consumption through tailored substance abuse programs for PWIDs. These initiatives should focus on reducing alcohol use and promoting harm-reduction practices to enhance adherence to ART. A collaborative approach in emphasizing family support is decisive and can educate and engage families about the importance of supporting PWIDs in adhering to their treatment. Support groups, counseling services, and educational campaigns can foster a supportive environment and promote higher levels of ART adherence. Moreover, governments and implementing partners should combat HIV-related stigma, encouraging individuals to disclose their status by implementing educational campaigns and training programs that can raise awareness about the benefits of ART and information disclosure among PWIDs, healthcare providers, and the community, leading to improved adherence rates. These comprehensive efforts are necessary to achieve the recommended adherence targets and enhance the overall health and well-being of PWIDs living with HIV.

### What is known about this topic

- *The level of adherence to ART among people who inject drug living with HIV in Tanzania and other developing countries is relatively low;*
- *Worldwide people who inject drug are among the key vulnerable population with high HIV transmission rate*.

### What this study adds

- *The study provides the most recent data on antiretroviral therapy (ART) adherence among people who inject drugs living with HIV in Tanzania;*
- *The study highlights the key factors associated with adherence to ART among people who inject drugs living with HIV, which will be targeted in Tanzania and other developing countries to improve ART adherence;*
- *This research adds to the ongoing scholarly discussion on this relevant topic*.

## References

[1] UNAIDS HIV and people who use drugs-human rights fact sheet series 2021.

[2] Sub-Saharan Africa harm reduction (2018). Regional Overview: 2.9 sub-Saharan Africa, Global State of Harm Reduction.

[3] UNAIDS (2020). Country progress report - United Republic of Tanzania. Global AIDS Monitoring.

[4] Tanzania National Bureau of Statistics (NBS) (2018). Tanzania HIV Impact Survey (This) 2016-2017.

[5] Joint United Nations Programme on HIV/AIDS (2018). Miles to go: closing gaps, breaking barriers, righting injustices: global AIDS update.

[6] Hansana V, Sanchaisuriya P, Durham J, Sychareun V, Chaleunvong K, Boonyaleepun S (2013). Adherence to antiretroviral therapy (ART) among people living with HIV (PLHIV): A cross-sectional survey to measure in Lao PDR. BMC Public Health.

[7] Semvua SK, Orrell C, Mmbaga BT, Semvua H, Bartlett JA, Boulle AA (2017). Predictors of non-adherence to antiretroviral therapy among HIV infected patients in northern Tanzania. PLoS One.

[8] Mbogo LW, Sambai B, Monroe-Wise A, Ludwig-Barron NT, Guthrie BL, Bukusi D (2022). Participation in methadone programs improves antiretroviral uptake and HIV viral suppression among people who inject drugs in Kenya. J Subst Abuse Treat.

[9] Ali B, Nisar N, Nawab F (2018). Adherence to antiretroviral therapy in HIV-positive, male intravenous drug users in Pakistan. East Mediterr Health J.

[10] Kizindo J, Marealle AI, Mutagonda R, Mlyuka HJ, Mikomangwa WP, Kilonzi M (2022). Adherence to Antiretroviral Therapy Among HIV-Infected Clients Attending Opioid Treatment Program Clinics in Dar es Salaam, Tanzania. Cureus.

[11] Chan PY, Joseph MA, Jarlais DCD, Uusküla A (2018). Perceived effectiveness of antiretroviral therapy, self-rated health and treatment adherence among HIV-positive people who inject drugs in Estonia. Int J STD AIDS.

[12] Culbert GJ, Waluyo A, Wang M, Putri TA, Bazazi AR, Altice FL (2019). Adherence to antiretroviral therapy among incarcerated persons with HIV: association with methadone and perceived safety. AIDS Behav.

[13] Lancaster KE, Mollan KR, Hanscom BS, Shook-Sa BE, Ha T V, Dumchev K (2021). Engaging people who inject drugs living with HIV in antiretroviral treatment and medication for opioid use disorder: extended follow-up of HIV Prevention Trials Network (HPTN) 074. Open Forum Infect Dis.

[14] Feelemyer J, Des Jarlais D, Arasteh K, Uusküla A (2015). Adherence to antiretroviral medications among persons who inject drugs in transitional, low and middle income countries: an international systematic review. AIDS Behav.

[15] Hoots BE, Finlayson TJ, Broz D, Paz-Bailey G (2017). Antiretroviral therapy use among HIV-infected people who inject drugs-20 cities, United States, 2009-2015. J Acquir Immune Defic Syndr.

